# Prevalence of *Clostridium difficile* Infection among Solid Organ Transplant Recipients: A Meta-Analysis of Published Studies

**DOI:** 10.1371/journal.pone.0124483

**Published:** 2015-04-17

**Authors:** Suresh Paudel, Ioannis M. Zacharioudakis, Fainareti N. Zervou, Panayiotis D. Ziakas, Eleftherios Mylonakis

**Affiliations:** Infectious Diseases Division, Warren Alpert Medical School of Brown University, Rhode Island Hospital, Providence, Rhode Island, United States of America; Cleveland Clinic, UNITED STATES

## Abstract

Several factors including antibiotic use, immunosuppression and frequent hospitalizations make solid organ transplant (SOT) recipients vulnerable to *Clostridium difficile* infection (CDI). We conducted a meta-analysis of published studies from 1991-2014 to estimate the prevalence of CDI in this patient population. We searched PubMed, EMBASE and Google Scholar databases. Among the 75,940 retrieved citations, we found 30 studies coded from 35 articles that were relevant to our study. Based on these studies, we estimated the prevalence of CDI among 21,683 patients who underwent transplantation of kidney, liver, lungs, heart, pancreas, intestine or more than one organ and stratified each study based on the type of transplanted organ, place of the study conduction, and size of patient population. The overall estimated prevalence in SOT recipients was 7.4% [95%CI, (5.6-9.5%)] and it varied based on the type of organ transplant. The prevalence was 12.7% [95%CI, (6.4%-20.9%)] among patients who underwent transplantation for more than one organ. The prevalence among other SOT recipients was: lung 10.8% [95% CI, (5.5%-17.7%)], liver 9.1 % [95%CI, (5.8%-13.2%)], intestine 8% [95% CI, (2.6%-15.9%)], heart 5.2% [95%CI, (1.8%-10.2%)], kidney 4.7% [95% CI, (2.6%-7.3%)], and pancreas 3.2% [95% CI, (0.5%-7.9%)]. Among the studies that reported relevant data, the estimated prevalence of severe CDI was 5.3% [95% CI (2.3%-9.3%)] and the overall recurrence rate was 19.7% [95% CI, (13.7%-26.6%)]. In summary, CDI is a significant complication after SOT and preventive strategies are important in order to reduce the CDI related morbidity and mortality.

## Introduction

*Clostridium difficile* is the most common cause of hospital-acquired infections [1]. Recent studies report the prevalence of *Clostridium difficile* infection (CDI) among hospitalized patients to be 0.9% [2]. Studies have reported an increase in hospitalization rates associated with *C*. *difficile* infection (CDI) [3]. There has also been a remarkable increase in mortality among CDI patients in hospitals [4], as mortality from CDI increased five-fold from 1999/2000-2005/2006 [5] and CDI accounts for≥ $4.8 billion in excess health-care costs [6].

Solid organ transplant (SOT) recipients are at high risk for CDI because of impaired defense mechanisms resulting from immunosuppression, perioperative antibiotic use and organ failure [7–9]. A study among 49,198 SOT recipients that used data from the 2009 US inpatient sample database noted that these patients are at greater risk for CDI compared to the general hospital population and estimated the prevalence of infection in SOT patients to be 2.7% [10]. However, this earlier study captured CDI episodes that occurred in an indeterminate time after transplantation and did not provide data for the initial hospitalization period post-transplantation where the most CDI episodes are expected to occur. In order to address these issues, we performed a meta-analysis to estimate the prevalence of CDI in SOT patients during the peri-transplant and post-transplantation period in university-based, tertiary medical centers. Also, we aimed to stratify the results based on type of the organ transplanted and estimate the recurrence rate and severity of CDI in this specific patient population.

## Materials and Methods

### Study Selection

We (S.P and I.M.Z) searched PubMed (1978 to February 2015), EMBASE and Google Scholar databases to identify studies that reported the prevalence of CDI among SOT recipients. The concise search term for PubMed was transplant* AND (clostrid* OR difficile OR diarrhea OR infect* OR (clostridium difficile) OR (pseudomembranous colitis)). The terms infect* and diarrhea were included in the search term in order to retrieve all articles that report episodes of CDI along with other infections, as well as episodes of CDI along with other causes of diarrhea in SOT patients. Articles that were considered eligible by title and abstract reading were assessed in full text. The reference lists of the eligible studies were also reviewed to find possible studies that match our search. Our meta-analysis follows the Preferred Reporting Items for Systematic Reviews and Meta-Analyses (PRISMA) guidelines (S1 Table) [11].

### Inclusion Criteria

Only studies that reported the prevalence of CDI among SOT patients during the peri-transplant period were included. Peri-transplant period was defined as “the time of transplant to the first discharge from the hospital” [12]. Studies that did not include follow-up of patients during the initial hospitalization post transplantation were excluded. Studies with adequate quality as described below in the *Quality Assessment* section were included. Also, studies published in a language other than English were excluded from the analysis.

### Outcomes of Interest

The primary outcome of interest of this meta-analysis was the prevalence of CDI among SOT patients. CDI was defined as “the presence of symptoms (usually diarrhea) and either a stool test result positive for *C*. *difficile* toxins or toxigenic *C*. *difficile* or colonoscopic findings demonstrating pseudomembranous colitis” [13]. Prevalence was calculated as the proportion of the patients diagnosed with CDI among the patients “at risk”, i.e. patients who received solid organ transplantation. A subgroup analysis was performed according to the type of organ transplanted, location of study conduction, and size of study population.

### Data Extraction

Studies that were considered for inclusion in the meta- analysis were evaluated by two reviewers (S.P. and I.M.Z.) and all relevant information from the text, figures, tables and charts were extracted for analysis. Studies that contained duplicate information were included only once. Extracted data include period of the study, patient population and location. The total number of patients who underwent SOT during the study period and total number of CDI cases among them were also extracted. The median follow-up duration, methods of *C*. *difficile* isolation, study design, number of severe cases, and number of recurrent episodes were also included. Recurrent CDI episodes were defined as onset of symptoms after complete abatement of symptoms with proper antibiotic therapy with additional positive CDI assays. Only data about the first CDI recurrence were used for the estimation of the recurrent rate in our study population. Severe cases were represented by cases that needed surgery for colitis and/or cases that required admission in the intensive care unit due to complications directly related to CDI and/or died from CDI-related cause [10].

### Quality Assessment

The methodological quality of eligible studies was assessed by two reviewers (S.P. and I.M.Z.) using the Newcastle-Ottawa Quality Assessment Scale, which is a ‘star based’ rating system [14]. The parameters used to evaluate the quality of individual studies were representativeness of the exposed cohort, ascertainment of exposure, demonstration that outcome of interest was not present at start of the study, assessment of outcome, follow-up long enough for outcomes to occur and adequacy of follow up of cohorts [15]. Two parameters ‘selection of the non-exposed cohort’ and ‘comparability of cohorts on the basis of the design or analysis’ were not applicable to our analysis, so each article could get up to 6 stars. We considered the study population representative of the exposed cohort if data on CDI were provided for all available transplant patients and not among a specific sub population. The outcome was assessed by the cases who presented with symptoms and laboratory diagnosis of CDI. Follow-up duration of at least 3 months was considered adequate for the outcome to occur in the cohort. Studies that received 5 stars were considered of adequate quality for extraction of relevant information.

### Statistical Analysis

We performed the meta-analysis using a random-effects model to estimate the pooled prevalence and the 95% confidence intervals (CI), using Der-Simonian and Laird weights [16]. The double arcsine methodology was used to avoid an undue large weight for studies with low or high prevalence (prevalence close to 0 or 1) [17]. The Egger’s test [18,19] was used to assess small study effects. Between-study variance τ^2^estimation [20] was used to assess statistical heterogeneity. Subgroup analyses were used to account for possible sources of heterogeneity. The meta and metareg commands of the Stata v13 software package (Stata Corporation, College Station, TX) were used to perform the statistical analysis. The statistical significance threshold was set at 0.05.

## Results

**Fig 1:**
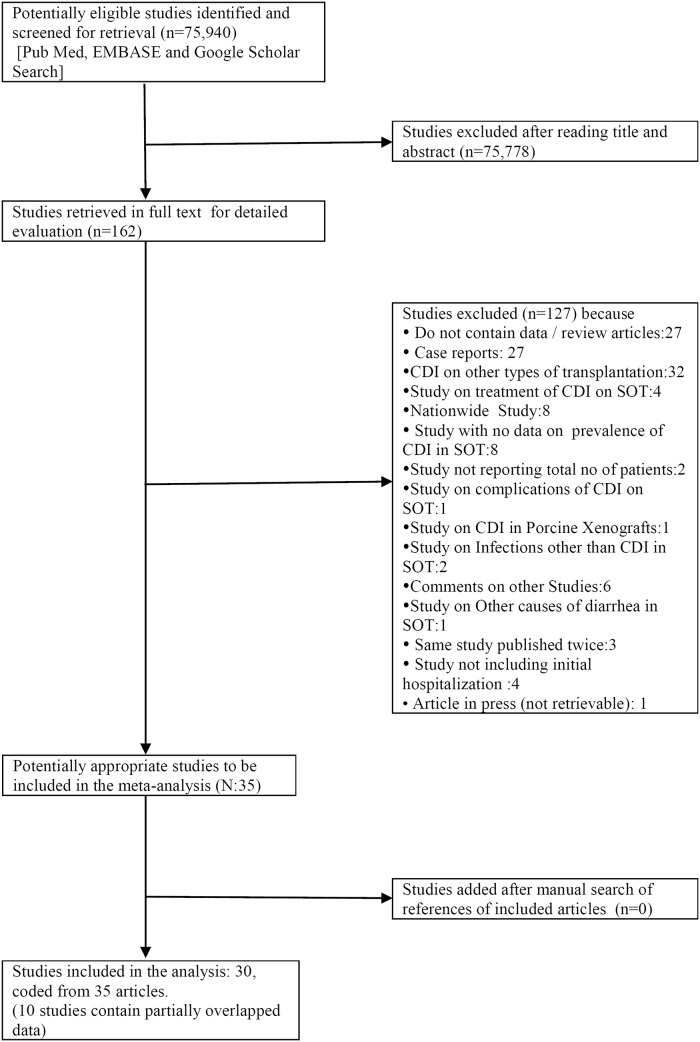
Flow chart of meta-analysis.

Our literature search yielded 75,940 citations and the last day of the literature search was February 9, 2015. After scrutinizing the titles and abstracts of retrieved articles, 162 articles were accessed in full text. Among these 162 articles, 127 studies were excluded because they did not contain extractable data on prevalence of CDI among SOT patients. Of the total 35 remaining eligible studies, 10 contained partially overlapping data [21–30] and data were extracted with an effort to extract the maximum available data. We included 30 studies in the final analysis coded from 35 articles. The details of the selection process of eligible articles are presented in the Flow chart (Fig 1).

The studies included in our analysis were published from 1991–2014 and reported data on 21,683 patients. We stratified the patients according to the type of organ transplanted. Among them 10,659 (49.2%) were kidney recipients, 5,433 (25.1%) liver, 1,556 (7.2%) lung, 1,397 (6.4%) heart, 539 (2.5%) pancreas, 60 (0.27%) intestine, 2 (0.01%) hand, 751 (3.5%) multiple-organs and 1508 (7%) were unspecified solid organ transplant recipients. Among the multiple-organ transplant recipients; combined kidney/pancreas transplant was by far the most common combination (n = 729, 97.1%). Data from individual studies are presented in Table 1 and all studies were considered of adequate quality on the basis of Newcastle-Ottawa scale (S2 Table).

**Table 1:** Individual Studies.

Author	Year	Origin	Population	Study Period	N (Transplant recipients)	n- CDI	Recurrence (No. of cases)	Severity (No. of cases)	Quality score
Mittal C[38]	2014	Henry Ford Hospital, Detroit, Michigan, U.S.A	Liver transplant patients	Jan 2000- Dec 2010	Li: 970	Li: 183	31	Colectomy:5	6
Hsu JL[31]	2014	University of Wisconsin, U.S.A	Kidney or Liver transplant patients	Jan 1994- Dec 2008	4666 (K:3630, Li:1036)	170 (K:107, Li:63)	29	ICU admission: 11 Colectomy:0	5
Tsapepas DS[27]	2014	Columbia University Medical Center	Single Solid organ transplant patients	Sep 2009- Dec 2012 (Median follow-up:23 months)	Li:331, Lu: 200, H:254	Li: 9, Lu: 14, H:5	4	ICU admission/ Colectomy: NR Mortality:1	6
Garg S[54]	2014	Johns Hopkins University, U.S.A	Liver transplant patients	Jan 2006-Jun 2013	Li: 251	Li: 32	9	NR	5
Dorschner P[55]	2014	Northwestern university Feinberg SoM, Chicago	Solid organ transplant patients	Jan 2012- Jul 2012 (Follow-up: 30 days)	146	1	NR	NR	5
Neofytos D[43]	2013	Johns Hopkins Hospital, U.S.A	Adult Kidney transplant patients	Jan 2008-Dec 2010	K:603	K: 37	4	Colectomy/ ICU admission: 1	5
Deshpande A[30]	2013	Cleveland Clinic, Ohio, U.S.A	Single Organ transplant patients	Jan 2003- Dec 2009	Lu: 525, Li: 809	Lu: 37, Li: 69	Discrete data on episodes only	Colectomy:2 Mortality: 0	6
Wheeler M[56]	2013	University of North Carolina	Solid organ transplant patients	2005–2012 (Follow-up: 1 year)	872	31	5	Colectomy: 0 Mortality: 0	6
Kittleson M[57]	2013	Sinai Heart Institute, CA, U.S.A	Adult Heart transplant patients	2000–2010 (Median follow-up: 1year)	H: 554	H:22	NR	NR	5
Lee JT[25]	2013	University of Minnesota, U.S.A	Lung transplant patients	Jan 2000-Mar 2011	Lu: 388	Lu:89	36	Total severe cases: 5 (Colectomy:3 Mortality:2	6
Shah SA [28]	2013	Columbia University, Weill Cornell Medical Center, U.S.A	Adult Kidney or Pancreas transplant recipients	Jan 2009- Feb 2011 (Median follow-up: 291 days)	998 (K: 942, P: 56)	28 (K:24, P:4)	8	NR	6
Boutros M [51]	2012	McGill University Health Center, Montreal, Canada	Single or Multi-Organ transplant patients	Jan 1999- Mar 2010	1331(K: 814, Li: 430, H: 112, P:109, K+P: 88, K+H:10, K+Li:7)	165 (K:92, Li: 90, H: 9, K+P:8)	14	Colectomy:6 ICU admission: 26 Mortality: 14	5
Ott E [58]	2011	Europe	Cardio-thoracic transplant patients	2007–2009	366	21	NR	Colectomy: 0	5
Abid S [50]	2011	University College of Medicine, U.S.A	Kidney or Liver transplant patients	2005–2009	K: 365, Li: 41	K:41, Li:6	NR	NR	6
Mitu- Pretorian OM [45]	2011	Manchester Royal Infirmary UK	Kidney and/or Pancreas transplant patients	Jan 2004- Dec 2007 (Follow up: 1 year)	682 (K:576, P:18, K+P: 88)	24 (K+P: 4, K: 20, P:0)	NR	Colectomy: 2	6
Rosen JB [52]	2010	Texas Children Hospital, U.S.A	Pediatric lung transplant patients	Oct 2002–2008	Lu:74	Lu: 4	2	Ileostomy: 1	5
Rostambeigi N [59]	2010	Mayo Clinic College of Rochester, MN, USA	Adult Pancreas and/or Kidney transplant patients	Jan 1998- July 2006 (Median follow-up: 6.4 years)	216 (K+P: 149, P: 67)	9 (K+P:6, P:3)	1	NR	5
Coltart IC [53]	2009	King’s College Hospital, UK	Liver transplant patients	Jan 2006- April 2007	Li: 191	Li: 31	NR	Colectomy: 0 Death: 0	5
Gunderson CC[46]	2008	Oschner Medical Center, Louisiana, USA	Lung transplant patients	Nov 1990- Nov 2005 (Median follow-up: 2.7 years)	Lu: 202	Lu:15	NR	Colectomy:2	6
Theunissen C[47]	2008	Erasame University Hospital, Belgium	Adult lung transplant patients with Cystic Fibrosis	Jan 1998- Dec 2004 (Median follow-up: 4.6 years)	Lu: 49	Lu: 16	NR	Severe cases: 5 (Surgery:2,Mortality:2	6
Stelzmueller I [21]	2007	Innsbruck Medical University, Austria	Single and Multi-organ transplant patients	Jan 1994-Dec 2005	2799 (K:1438, Li:651, P:289, H:242, Lu:118, H+Lu:5, I:27,Islet:25,Hand:2)	36 (K:4, L:20, P:2, H:3, Lu:4, H+Lu:5, Intes:2, Hand:1)	NR	Colectomy: 2	5
Munoz P [32]	2007	University of Madrid, Spain	Heart transplant patients	Jan 1993- Dec 2005	H: 235	H: 35	10	Colectomy: 0	6
Hashimoto M [60]	2007	Tokyo University Hospital, Japan	Adult Liver transplant patients	Jan 1996-Nov 2004	Li: 242	Li: 11	2	NR	5
Albright JB [61]	2007	Mayo Clinic, Jacksonville, Florida U.S.A	Liver transplant patients (cadaveric grafts)	Mar 1998-Dec 2001	Li: 402	Li: 32	7	Colectomy:0	6
Michalak G [62]	2005	University of Warsaw, Poland	Patients with Diabetes and end stage renal disease with Kidney and Pancreas transplant	1998–2004	K+P:51	K+P:8	NR	NR	6
Ziring D [63]	2005	David Geffen School of Medicine at UCLA, CA, U.S.A	Intestine transplant patients	Nov 1991-May 2003 (Median follow-up: 12 months)	I:33	I:2	NR	NR	6
Keven K [48]	2004	University of Pittsburgh Medical Center, U.S.A	Adult Kidney and/or Pancreas transplant patients	Jan 1999-Dec 2002	702 (K: 600, K+P: 102)	35 (K: 27, K+P: 8)	8 (K:7, K+P:1)	Colectomy:2 (1 death among them)	6
Loinaz C [64]	2003	University of Miami, School of Medicine, Florida, U.S.A	Adult and Pediatric Intestine, Liver/Intestine or Multi visceral transplant patients	1994–2001	124	3	NR	NR	6
West M[49]	1999	University of Minnesota, U.S.A	Adult and Pediatric Kidney and/or Pancreas transplant patients	Jan 1985-Dec 1994	1932 (Pediatric K:267; Adult K:1424, K+P: 251)	159 (Pediatric K: 43, Adult K:50, K+P:39)	6	No severe cases	6
George DL[65]	1991	University of Chicago, U.S.A	Patients receiving orthotropic Liver transplant	Feb 1985- July 1987 (Median follow-up: 324 days)	Li: 79	Li: 2	NR	NR	6

**Footnotes**: H: Heart, I: Intestine, ICU: Intensive Care Unit, K: Kidney, Li: Liver, Lu: Lungs, NR: Not reported, P: Pancreas.

Geographical location varied among the included articles. Among the 30 studies, 22 were conducted in the North America, 7 in Europe, and 1 in Asia. All studies were retrospective. Twenty one studies reported the method of isolation of *C*. *difficile*. One or more than 1 method of isolation such as stool culture and toxin detection were used in the studies. *C*. *difficile* toxin was identified using one or more of the following methods: enzyme immunoassay, radio immunoassay, tissue culture assay and PCR. The method of *C*. *difficile* isolation used in each individual study is included in Table 2.

**Table 2:** Individual Studies.

Author	Year	Follow-up	Recurrence (No. of cases)	Recurrence (definition)	Method of diagnosis	Time of CDI diagnosis
Mittal C	2014	NR	31	New onset of diarrhea or positive stool toxin assay within 12 weeks of CDI	Until2008: EIA for toxin A/B, From 2009: Glutamate dehydrogenase followed by EIA and molecular testing	Mean: 51 days
Hsu JL	2014	NR	29	Episodes of CDI<8 weeks after resolution of symptoms from previous episode	Positive CD culture and stool toxin, pseudomembranous colitis on endoscopy/ histopathology	Mean: 653 days
Tsapepas DS	2014	Median follow-up: 23 months (16–31 months)	4	Resurgence of diarrhea after the cessation of initial therapy, confirmed by a subsequent stool specimen with detection of *C*. *difficile* toxin B by PCR	Toxin detection by PCR	51 days
Garg S	2014	NR	9	More than 1 episode of CDI in an OLT recipient at any time after OLT	Cytotoxin assay or PCR for toxin gene B	NR
Dorschner P	2014	30 days	NR	NR	NR	NR
Neofytos D	2013	6 months	4	New episode of CDI after at least14 completed days of treatment for primary CDI	Cytotoxin assay for toxins A and B or PCR	Mean: 9 days
Deshpande A	2013	NR	Data on recurrent episodes	CDI diagnosed using the same criteria as for the first episode and occurring after an initial CDI episode has resolved completely with treatment	Toxin detection by EIA	Median:116 days (Lung transplant patients),23 days (Liver transplant patients)
Wheeler M	2013	1 year	5	Reappearance of CDI within 2 months	Not Specified	26 days
Kittleson M	2013	1 year	1	NR	NR	NR
Lee JT	2013	Median follow-up: 4.2 years	36	Recurrence of symptoms with positive CDI assay after complete abatement of symptoms with antibiotics	Until 2007: Stool culture and toxin assay with EIA, From 2007: Toxin assay and PCR	177 days (4days-6.9 years)
Shah SA	2013	Median follow-up: 291 days	8	Recurrence of symptoms with positive CD PCR between 6–50 days after stopping treatment	Gene B PCR	Median: 57 days
Boutros M	2012	NR	14	Two clinical episodes of CDI(with positive cytotoxin assay) more than two months apart	Cell culture cytotoxin assay for toxin B	NR
Ott E	2011	NR	NR	NR	NR	NR
Abid S	2011	NR	NR	NR	NR	NR
Mitu-Pretorian OM	2011	NR	NR	NR	Stool culture and toxin assay	12.5 days (3days-90 days)
Rosen B	2010	NR	2	NR	Toxin assay and PCR	NR
Rostambeigi N	2010	6.4 years	1	NR	NR	NR
Coltart IC	2009	90 days	NR	NR	Stool toxin assay	15.5 days
Gunderson CC	2008	2.7 years	NR	NR	CD toxin A assay	NR
Theunissen C	2008	4.6 years	NR	NR	Stool culture, Detection of cytotoxin by tissue culture	NR
Stelzmueller I	2007	NR	NR	NR	Until 1995:Stool culture and toxin assay with RIA; From 1996: Stool culture and toxin assay with EIA	NR
Munoz P	2007	50 days	10	NR	Stool culture and CD toxin B by cell culture cytotoxin test	Mean: 32 days
Hashimoto M	2007	3 months	2	NR	Stool culture, ICA for CD toxin A, latex test for CD protein glutamate dehydrogenase	Mean: 19 days
Albright JB	2007	5 days-1999 days	7	NR	EIA for toxin A and B	NR
Michalak G	2005	NR	NR	NR	NR	NR
Ziring D	2005	12 months (2 months-69 months)	NR	NR	CD toxin Immunoassay	NR
Keven K	2004	NR	8	NR	CD toxin assay	30 days
Loinaz C	2003	535±58.12 days	NR	NR	NR	NR
West M	1999	NR	6	NR	Stool culture or CD toxin assay	NR
George DL	1991	324 days (70 days- 883 days)	NR	NR	NR	NR

**Footnotes:** CD: *Clostridium difficile*, CDI: *Clostridium difficile* infection, EIA: enzyme immunoassay, ICA: immune chromatographicassay, NR: not reported, PCR: polymerase chain reaction, RIA: radio immunoassay

**Fig 2:**
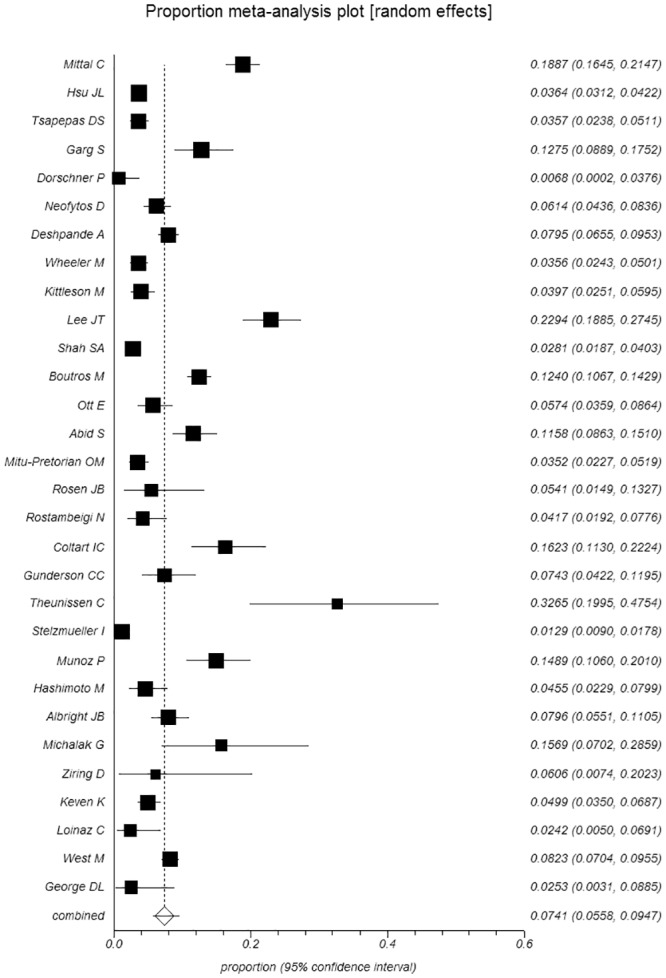
Prevalence of CDI among solid organ transplant recipients.

The pooled prevalence of CDI among 21,683 SOT recipients was 7.4% [95%CI, (5.6–9.5%), τ^2^ = 0.039] (Fig 2). According to the Egger’s test, we found no evidence of small study effects for the overall estimated prevalence (bias: 1.93, p-value: 0.06). We also stratified our data based on the type of organ transplanted. The prevalence of CDI inpatients who underwent transplantation for more than one organ was 12.7% [95%CI, (6.4%-20.9%), τ^2^ = 0.069] (S1 Fig). The prevalence among other single SOT recipients was: lung transplant recipients 10.8% [95%CI, (5.5%-17.7%), τ^2^ = 0.064] (S2 Fig), liver 9.1% [95%CI, (5.8%-13.2%), τ^2^ = 0.048] (S3 Fig), intestine 8% [95% CI, (2.6%-15.9%), τ^2^ = 0.00] (S4 Fig), heart 5.2% [95%CI, (1.8%-10.2%), τ^2^ = 0.042] (S5 Fig), kidney 4.7% [95% CI, (2.6%-7.3%), τ^2^ = 0.028] (S6 Fig) and pancreas 3.2% [95% CI, (0.5%-7.9%), τ^2^ = 0.030] (S7 Fig). The differences in prevalence between kidney and liver (p-value: 0.07) and kidney and lung (p-value: 0.07) were marginally significant. The prevalence of CDI among kidney transplant recipients was also lower than intestine, heart and multiple organ recipients, but these differences did not reach statistical significance [kidney-heart (p-value: 0.83), kidney-intestine (p-value: 0.48) and kidney-multiple organs (p-value: 0.13)].

The estimated prevalence of CDI in SOT patients was 6.8% [95% CI (5.0%-9.0%), τ^2^ = 0.032] from studies conducted in North America and 10.5% [(4.9%-18.0%), τ^2^ = 0.076] from studies conducted in Europe. We also stratified the studies based on population size (<200 versus >200) and we did not find significant difference in the estimated prevalence of CDI in SOT recipients (p-value: 0.65) (Table 3).

**Table 3:** Summary Estimates.

CDI	Studies (arms)	N	Combined Effect (95% CI)	τ^2^	P-value
**All Studies**	30 (35)	21,683	7.4% [95%CI, (5.6–9.5%)]	0.039	
Kidney	9	10,659	4.7% [95% CI (2.6%-7.3%)]	0.028	Ref
Liver	12	5,433	9.1% [95% CI (5.8%-13.2%)]	0.048	0.07
Lungs	7	1,556	10.8% [95% CI (5.5%-17.7%)]	0.064	0.07
Heart	6	1,397	5.2% [95% CI (1.8%-10.2%)]	0.042	0.83
Pancreas	4	539	3.2% [95% CI (0.5%-7.9%)]	0.030	0.54
Intestine	2	60	8.0% [95% CI (2.6%-15.9%)]	0.000	0.48
Multiple Organ	7	751	12.7% [95% CI (6.4%-20.9%)]	0.069	0.13
**Geographical region**					
North America	22	15,737	6.8% [95% CI (5.0%-9.0%)]	0.032	
Europe	7	5,704	10.5% [95% CI (4.9–18.0%)]	0.076	
**Populations**					
≥200 patients	22	20,936	7.1% [95% CI (5.2%-9.4%)]	0.037	Ref
<200 patients	8	747	8.6% [95% CI (3.2%-16.1%)]	0.099	0.65

Fifteen studies reported data on recurrence of CDI among 1,020 infected patients. Among them, 10 studies included the definition of recurrence. Three studies defined an episode as recurrent when the diagnosis was done within 8 weeks from the initial episode, 2 had a time frame longer than 8 weeks and the remaining 5 did not specify the duration (Table 2). Those studies that used a longer follow-up period to define recurrent episodes tend to overestimate the rate of recurrence. The reported recurrence rate was estimated to be 19.7% [95% CI, (13.7%-26.6%), τ^2^ = 0.073]. Definition of recurrence, time to recurrence, mean time to diagnosis and methods of isolation of *C*. *difficile* for individual studies are presented in the Table 2. Finally the reported prevalence of severe CDI from 19 studies was 5.3% [95% CI (2.3%-9.3%), τ^2^ = 0.097] and among the 1,243 *C*. *difficile* infected patients that were included in these studies there were 27 reported cases of CDI related colectomy, 37 patients had ICU admission and 20 patients died due to CDI related complications. The prevalence of colectomy due to CDI related complications in our study was 2.7% [95% CI (1.3%-4.6%), τ^2^ = 0.023]. Due to limited data on individual cases that required ICU admission and/or died, statistical representation could not be done in our analysis.

## Discussion

*C*. *difficile* infection has been increasingly recognized among SOT recipients [31]. The estimated prevalence of CDI in SOT patients in our study was 7.4%, which is higher than the 0.9% reported in the general hospital population [2]. In our study, lung and liver transplant patients had higher prevalence of CDI compared to kidney transplant recipients and this difference was marginally significant. There was no significant difference in the prevalence based on the size of the study population. Recurrence of CDI episodes was seen in 19.7% of cases and 5.3% of patients had severe CDI resulting in colectomy, ICU admission or death.

The overall estimated prevalence of CDI in our study is almost 3 times higher than that reported in a previous study which used data from 2009 nationwide inpatient sample database (2.7%) [10]. The fact that we included data from the initial hospitalization after transplantation might be the reason for the higher overall estimated prevalence. Indeed, most cases of CDI among SOT recipients are diagnosed in the early post-transplantation period due to intense immunosuppression, more frequent antimicrobial exposure and increased exposure to the health care setting [7]. The observed difference might also be due to the long duration of follow-up in the studies included in our meta- analysis as the follow-up time in most of our studies was up to several months and even years after transplantation.

In agreement to the study that reported the data from nationwide inpatient sample database [10], the prevalence of CDI was higher among lung transplant recipients in comparison to the kidney transplant patients. This might be attributed to the higher level of immunosuppression required for lung transplant patients [24]. Also, lung transplant patients often have a history of prolonged exposure to antibiotics and frequent hospital admissions, increasing their risk for CDI [25]. Also, patients who underwent transplantation of more than one organ had more than 3-times higher point prevalence of CDI than kidney recipients in our study, but this difference did not reach statistical significance, possibly due to the limited number of cases of multi-organ transplant patients.

Recurrence is a common problem in SOT patients with CDI [32]. Recurrent CDI can occur either because of relapse or because of re-infection; the relative frequency of each of these mechanism has not been well described [33]. Risk factors, such as increased length of hospital stay, prolonged use of antibiotics, immunosuppression and co-morbid conditions are associated with recurrence of CDI in SOT recipients [31,34] and, the recurrence rate varies based on the treatment of the initial CDI episode [35]. The estimated recurrence rate of CDI in our study was 19.7%, and it was comparable to the median 21.6% that was recently reported in a relevant meta-analysis in the general hospital setting [36].

Knowledge on the outcomes of CDI in SOT recipient is remarkably limited [37]. Few studies have reported an increase in the in hospital mortality, a longer hospital stay and cost of health care services among this group of patients [10,38]. CDI can also result in complications requiring colectomy and ICU admission [10]. Among the studies that reported data on severity, at least 5.3% of the patients had complications related to CDI in compared to 3%-5% rate of fulminant colitis in total hospitalized *C*. *difficile* infected patients [9,39,40]. The prevalence of colectomy in SOT patients due to CDI related complications in our study was 2.7% compared with 0.7% reported from the national inpatient sample database analysis in the general hospital population from 2001–2010 [41].

To locate possible sources of heterogeneity, we performed a subgroup analysis based on the size of the study population. Studies with small population size tend to overestimate the outcome. To evaluate this effect we stratified the studies based on population size (≥200 versus <200). The size of study population, however, did not significantly alter the estimated prevalence of CDI in SOT recipients (Table 3).

Notably, there were certain limitations in our study. Most of the studies that were included in our meta-analysis had a long study period (>6 years) and did not report the prevalence of CDI stratified by year. Therefore, it was not feasible to estimate the trend over time, so it is likely that the number of CDI cases in the recent times can be even higher. The follow-up period varied widely among studies included in our analysis making a sub-analysis of the data based on follow-up duration not possible. However, out of 13 studies that reported the mean time to CDI diagnosis, only 3 had a mean time of more than 3 months with the remaining 10 studies having a mean time to diagnosis within 3 months, supporting that most of the recorded episodes of CDI occurred shortly after SOT. Detection methods used to diagnose CDI among the studies included in our analysis differ in sensitivity [42]. Two or more methods of isolation of *C*. *difficile* were used in most of the studies and during different time frames, but the studies did not provide stratified extractable data. Therefore, a sensitivity analysis based on the diagnostic methods used was not feasible. Also, the definition of recurrence varied between studies [24,31,38,43] and in 2 of them the time to relapse of symptoms used for defining recurrent cases was longer than the 8 weeks used in current clinical practice guidelines for *Clostridium difficile* infection in adults [44]. The latter studies tend to overestimate the number of recurrent cases and therefore the estimated prevalence of recurrence in our study might not be a precise estimate as per the current clinical practice guidelines. The estimated recurrence rate in our study is a pooled data from studies using different treatment modalities, so different rate of treatment failure and recurrence might exist in each individual study. Twenty studies in our analysis mentioned possible risk factors associated with CDI in SOT recipients. They described prolonged exposure to antibiotics [27,43,45–50], immunosuppression [21,46,48–51] and increased days of hospitalization [26,27,38,43,45,46,50,52,53] to be associated with CDI. However, due to lack of raw data on each risk factor, statistical representation could not be performed in our study. Importantly, infection control measures and local epidemiology have a significant role in the prevalence of CDI among individual centers and our pooled estimation does not decrease the need of each local center to know the local prevalence.

In conclusion, our analysis estimated the pooled prevalence of CDI among SOT recipients to be almost 3 times higher than previously indicated. The observed high prevalence of CDI, along with the significant rate of severe cases, highlights the need for preventive policies, such as antimicrobial stewardship programs, strict compliance with hand hygiene and environmental decontamination that specifically target this patient population. Also, studies are needed to identify immunosuppressive and prophylactic antimicrobial regimens that are probably associated with lower risk of CDI.

## Supporting Information

S1 Fig Prevalence of CDI among multiple solid organ transplant recipients. (TIF)

S2 Fig Prevalence of CDI among lung transplant recipients. (TIF)

S3 Fig Prevalence of CDI among liver transplant recipients. (TIF)

S4 Fig Prevalence of CDI among intestine transplant recipients. (TIF)

S5 Fig Prevalence of CDI among heart transplant recipients. (TIF)

S6 Fig Prevalence of CDI among kidney transplant recipients. (TIF)

S7 Fig Prevalence of CDI among pancreas transplant recipients. (TIF)

S1 Table PRISMA checklist. (PDF)

S2 Table Quality Assessment. (PDF)
