# Supplementary figures and images for: Prevalence of Clostridium difficile Infection among Solid Organ Transplant Recipients: A Meta-Analysis of Published Studies
Source: PLoS One. 2015 Apr 17;10(4):e0124483. doi: 10.1371/journal.pone.0124483 (PMC4401454; doi:10.1371/journal.pone.0124483)

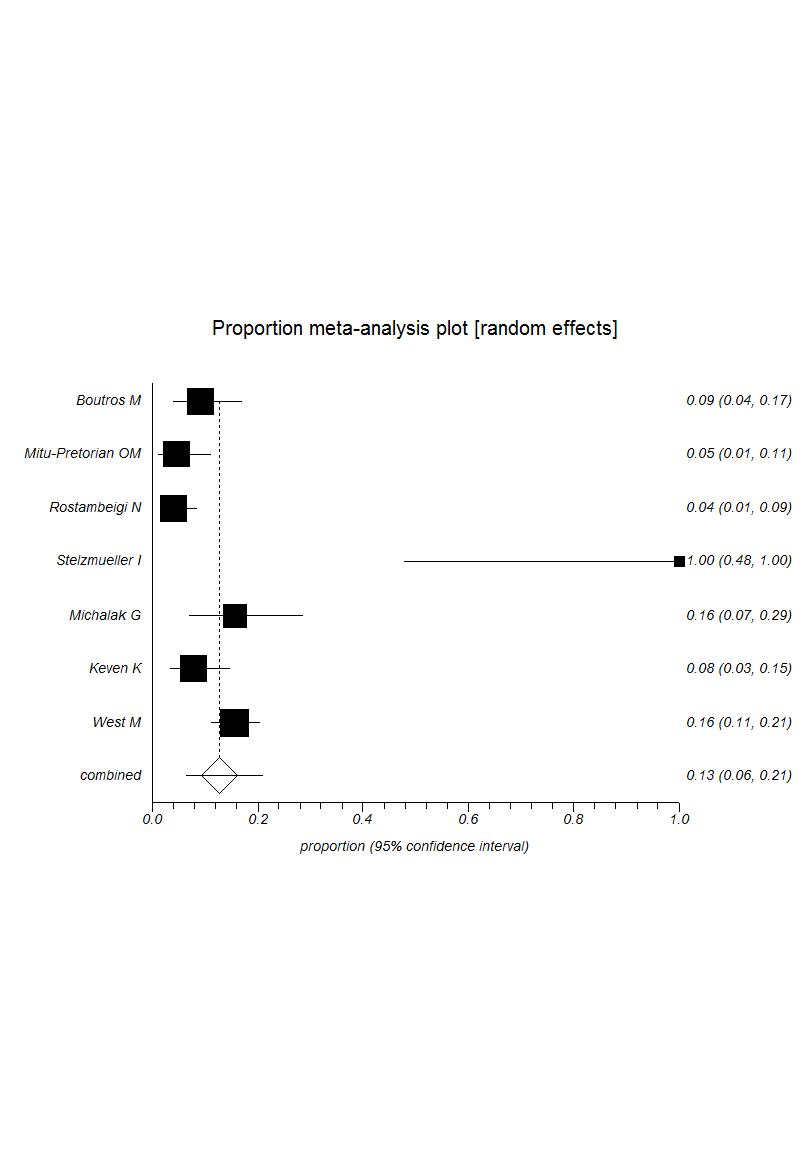

Supplement: S1 Fig — (TIF) [file pone.0124483.s001.tif]
