# Supplementary material for: Prevalence of Clostridium difficile Infection among Solid Organ Transplant Recipients: A Meta-Analysis of Published Studies
Source: PLoS One. 2015 Apr 17;10(4):e0124483. doi: 10.1371/journal.pone.0124483 (PMC4401454; doi:10.1371/journal.pone.0124483)
Supplement: S2 Table — (PDF) [file pone.0124483.s009.pdf]

S2 Table. Quality Assessment

Quality assessment criteria	Mittal C	Hsu JL	Tsapepas DS	Garg S	Dorschner P	Neofytos D	Deshpande A	Wheeler M
**Selection**								
Representativeness of exposed cohort?	*	*	*	*	*	*	*	*
Selection of the non-exposed cohort?	N/A	N/A	N/A	N/A	N/A	N/A	N/A	N/A
Ascertainment of exposure?	*	*	*	*	*	*	*	*
Demonstration that outcome of interest was not present at start of study?	*	*	*	*	*	*	*	*
**Comparability**								
Comparability of cohorts on the basis of the design or analysis	N/A	N/A	N/A	N/A	N/A	N/A	N/A	N/A
**Outcome**								
Assessment of outcome	*	*	*	*	*	*	*	*
Follow-up long enough for outcomes to occur	*		*				*	*
Adequacy of follow-up of cohorts	*	*	*	*	*	*	*	*
**Score**	**6**	**5**	**6**	**5**	**5**	**5**	**6**	**6**

Quality assessment criteria	Kittleson M	Lee JT	Shah SA	Boutros M	Ott E	Abid S	Mitu-Pretorian OM
**Selection**							
Representativeness of exposed cohort?	*	*	*	*	*	*	*
Selection of the non-exposed cohort?	N/A	N/A	N/A	N/A	N/A	N/A	N/A
Ascertainment of exposure?	*	*	*	*	*	*	*
Demonstration that outcome of interest was not present at start of study?	*	*	*	*	*	*	*
**Comparability**							
Comparability of cohorts on the basis of the design or analysis	N/A	N/A	N/A	N/A	N/A	N/A	N/A
**Outcome**							
Assessment of outcome	*	*	*	*	*	*	*
Follow-up long enough for outcomes to occur		*	*			*	*
Adequacy of follow-up of cohorts	*	*	*	*	*	*	*
**Score**	**5**	**6**	**6**	**5**	**5**	**6**	**6**

**Quality assessment criteria**	**Rosen JB**	**Rostambeigi N**	**Coltart IC**	**Gunderson CC**	**Theunissen C**	**Stelzmueller I**	**Munoz P**

Selection

Representativeness of exposed cohort?	*	*	*	*	*	*	*
Selection of the non-exposed cohort?	N/A	N/A	N/A	N/A	N/A	N/A	N/A
Ascertainment of exposure?	*	*	*	*	*	*	*
Demonstration that outcome of interest was not present at start of study?	*	*	*	*	*	*	*

Comparability

Comparability of cohorts on the basis of the design or analysis	N/A	N/A	N/A	N/A	N/A	N/A	N/A

Outcome

Assessment of outcome	*	*	*	*	*	*	*
Follow-up long enough for outcomes to occur				*	*		*
Adequacy of follow-up of cohorts	*	*	*	*	*	*	*

**Score**	**5**	**5**	**5**	**6**	**6**	**5**	**6**

**Quality assessment criteria**	**Hashimoto M**	**Albright JB**	**Michalak G**	**Ziring D**	**Keven K**	**Loinaz C**	**West M**	**George DL**
**Selection**								
Representativeness of exposed cohort?	*	*	*	*	*	*	*	*
Selection of the non-exposed cohort?	N/A	N/A	N/A	N/A	N/A	N/A	N/A	N/A
Ascertainment of exposure?	*	*	*	*	*	*	*	*
Demonstration that outcome of interest was not present at start of study?	*	*	*	*	*	*	*	*
**Comparability**								
Comparability of cohorts on the basis of the design or analysis	N/A	N/A	N/A	N/A	N/A	N/A	N/A	N/A
Outcome								
Assessment of outcome	*	*	*	*	*	*	*	*
Follow-up long enough for outcomes to occur		*	*	*	*	*	*	*
Adequacy of follow-up of cohorts	*	*	*	*	*	*	*	*
**Score**	**5**	**6**	**6**	**6**	**6**	**6**	**6**	**6**
